# Distinct genetic difference between the Duffy binding protein (PkDBPαII) of *Plasmodium knowlesi* clinical isolates from North Borneo and Peninsular Malaysia

**DOI:** 10.1186/s12936-015-0610-x

**Published:** 2015-02-21

**Authors:** Mun-Yik Fong, Sarah AA Rashdi, Ruhani Yusof, Yee-Ling Lau

**Affiliations:** Department of Parasitology, Faculty of Medicine, University of Malaya, 50603 Kuala Lumpur, Malaysia; Tropical Infectious Diseases Research and Education Centre (TIDREC), Faculty of Medicine, University of Malaya, 50603 Kuala Lumpur, Malaysia

**Keywords:** *Plasmodium knowlesi*, North Borneo, Duffy binding protein, Diversity, Selection, Haplotypes, Allle groups

## Abstract

**Background:**

*Plasmodium knowlesi* is one of the monkey malaria parasites that can cause human malaria. The Duffy binding protein of *P. knowlesi* (PkDBPαII) is essential for the parasite’s invasion into human and monkey erythrocytes. A previous study on *P. knowlesi* clinical isolates from Peninsular Malaysia reported high level of genetic diversity in the PkDBPαII. Furthermore, 36 amino acid haplotypes were identified and these haplotypes could be separated into allele group I and allele group II. In the present study, the PkDBPαII of clinical isolates from the Malaysian states of Sarawak and Sabah in North Borneo was investigated, and compared with the PkDBPαII of Peninsular Malaysia isolates.

**Methods:**

Blood samples from 28 knowlesi malaria patients were used. These samples were collected between 2011 and 2013 from hospitals in North Borneo. The PkDBPαII region of the isolates was amplified by PCR, cloned into *Escherichia coli*, and sequenced. The genetic diversity, natural selection and phylogenetics of PkDBPαII haplotypes were analysed using MEGA5 and DnaSP ver. 5.10.00 programmes.

**Results:**

Forty-nine PkDBPαII sequences were obtained. Comparison at the nucleotide level against *P. knowlesi* strain H as reference sequence revealed 58 synonymous and 102 non-synonymous mutations. Analysis on these mutations showed that PkDBPαII was under purifying (negative) selection. At the amino acid level, 38 different PkDBPαII haplotypes were identified. Twelve of the 28 blood samples had mixed haplotype infections. Phylogenetic analysis revealed that all the haplotypes were in allele group I, but they formed a sub-group that was distinct from those of Peninsular Malaysia. Wright’s F_ST_ fixation index indicated high genetic differentiation between the North Borneo and Peninsular Malaysia haplotypes.

**Conclusions:**

This study is the first to report the genetic diversity and natural selection of PkDBPαII of *P. knowlesi* from Borneo Island. The PkDBPαII haplotypes found in this study were distinct from those from Peninsular Malaysia. This difference may not be attributed to geographical separation because other genetic markers studied thus far such as the *P. knowlesi* circumsporozoite protein gene and small subunit ribosomal RNA do not display such differentiation. Immune evasion may possibly be the reason for the differentiation.

**Electronic supplementary material:**

The online version of this article (doi:10.1186/s12936-015-0610-x) contains supplementary material, which is available to authorized users.

## Background

*Plasmodium knowlesi*, a malaria parasite of macaque monkeys, was reported to cause a large number of human infections in the Malaysian state of Sarawak, North Borneo, in 2004 [1]. Subsequent to this landmark report, human knowlesi malaria cases have been documented in in other parts of Borneo Island, Peninsular Malaysia, and in many other countries in Southeast Asia [2]. In Malaysia, *P. knowlesi* has now overtaken *Plasmodium vivax* as the main cause of human malaria [3].

The invasion of a malaria parasite into its host erythrocyte depends on the interaction between the parasite’s protein and its corresponding receptor on the surface of the erythrocyte. *Plasmodium knowlesi* uses the Duffy blood group antigen as a receptor to invade erythrocytes [4]. The Duffy binding proteins of *P. knowlesi* (PkDBP) are located on their merozoites and occur as three distinct forms: α, β and γ. These are large proteins and each can be divided into seven regions (I-VII). Region II contains the critical motifs for binding to the erythrocyte. Region II of PkDBPα (designated as PkDBPαII) binds to Duffy-positive human erythrocytes and macaque erythrocytes. PkDBPβII and PkDBPγII, however, bind only to macaque erythrocytes and not to the Duffy antigen of human erythrocytes [5].

It has been observed that antibodies raised against PkDBPαII could inhibit *P. knowlesi* invasion of human and macque erythrocytes *in vitro* [6]. Therefore, like PvDBPII for vivax malaria, PkDBPαII may be a candidate vaccine antigen against knowlesi malaria. Any design of vaccine against malaria must take into consideration the nature and genetic polymorphism of the candidate antigen. In a recent study, a high level of genetic diversity was found in the PkDBPαII of 20 *P. knowlesi* clinical isolates from Peninsular Malaysia [7]. At the amino acid level, 36 haplotypes were identified and these haplotypes could be separated into allele group I and allele group II. In the present study, the PkDBPαII of clinical isolates from the Malaysian states of Sabah and Sarawak in North Borneo was investigated.

## Methods

### Blood samples

The 28 human blood samples used in this study were collected from knowlesi malaria patients at government hospitals in Sabah (n = 16) and Sarawak (n = 12) (Table 1). Confirmation of *P. knowlesi* infection in all the samples was carried out by microscopic examination of Giemsa-stained blood smears and nested polymerase chain reaction (PCR). Ethical approval for the use of human and monkey blood samples in this study were granted by the University of Malaya Medical Centre Ethic Committee (MEC No. 817.18) and the Medical Research Ethic Committee (MREC), Ministry of Health, Malaysia (National Medical Research Register ID No. 13079).

**Table 1:** **PkDBPαII haplotypes detected in the patient blood samples from Sabah (SBH) and Sarawak (SWK), North Borneo**

**Blood sample**	**Origin of sample (district)**	**Haplotype**
SBH1	Kudat	H37, H38, H39, H40
SBH2	Ranau	H41, H42, H43
SBH3	Ranau	H44
SBH4	Kota Kinabalu	H45, H46
SBH5	Kudat	H47, H48, H49, H50
SBH6	Kota Kinabalu	H47, H51, H52
SBH07	Kota Kinabalu	H53
SBH08	Kota Kinabalu	H54
SBH21	Kudat	H55
SBH31	Ranau	H47
SBH37	Ranau	H47, H56, H57
SBH47	Kudat	H58
SBH51	Kudat	H47, H59
SBH62	Ranau	H47, H60, H61
SBH68	Ranau	H47
SBH71	Ranau	H62
SWK01	Kuching	H63, H64
SWK07	Kuching	H42
SWK21	Kapit	H47
SWK24	Kapit	H65, H66
SWK46	Sri Aman	H67
SWK58	Kapit	H68
SWK59	Kapit	H69
SWK72	Kuching	H47
SWK76	Kapit	H70, H71
SWK86	Kapit	H72
SWK93	Kapit	H47, H73
SWK94	Kapit	H74

### Extraction of DNA

Total DNA of the *P. knowlesi* was extracted from each blood sample using the QIAGEN Blood DNA Extraction kit (QIAGEN, Hilden, Germany). In each extraction, 100 μl of blood was used. The extracted DNA was suspended in water to a final volume of 50 μl.

### PCR, cloning and sequencing of the PkDBPαII

The PkDBPαII was amplified by nested PCR using oligonucleotide primers Pkα-DBP-F1: 5′-CGCATTTTGAAGGAATCCAC-3′ and Pkα-DBP-R1: 5′-TGCTAGACTTACCTTCACCT-3′ for nest 1. The primers for the nest 2 reaction were Pkα-DBP-F: 5′-TCCTCAAAAGGCGGTGACCATCC-3′ and Pkα-DBP-R: 5′-ACTGGCTGCCTTAGATTCAACACCA-3′. Cycling conditions for nest 1 were as follows: 95°C for 4 min, 30 cycles at 95°C for 30 sec, 48°C for 30 sec, and 72°C for 90 sec, followed by a 10-min extension at 72°C. The amplification for nest 2 was performed using the following cycling profile: 95°C for 4 min, 30 cycles at 95°C for 30 sec, 56°C for 30 sec, and 72°C for 90 sec, followed by a 10-min extension at 72°C. The PCR product with an expected size of 1,053 bp was analysed on a 1% agarose gel.

### Purification of PCR products and DNA cloning

PCR products were purified by QIAquick PCR purification Kit (QIAGEN, Hilden, Germany) following the manufacturer’s instructions. The purified PCR products were then ligated into cloning vector pGEM-T® (Promega Corp, USA) and transformed into *Escherichia coli* TOP10F’. Plasmids of recombinant clones harbouring the PkDBPαII fragment were sent to a commercial laboratory for DNA sequencing. To detect possibility of multiple haplotypes infecting a patient, plasmids from four to six recombinant clones from each transformation mixture were sequenced.

### Analysis of PkDBPαII sequences

Multiple sequence alignment of PkDBPαII was performed using CLUSTAL-Omega programme which was available on-line [8]. Both nucleotide and the deduced amino acid sequences were aligned and analysed. Phylogenetic tree was constructed using the Neighbour Joining method described in MEGA5 [9]. In constructing the phylogenetic tree, bootstrap replicates of 1,000 were used to test the robustness of the tree.

### PkDBPαII sequence polymorphism analysis

DnaSP ver. 5.10.00 [10] was used to perform polymorphism analysis on the PkDBPαII sequences. Information such as the number of segregating sites (S), haplotype diversity (Hd), nucleotide diversity (π), and average number of pair-wise nucleotide differences within the population (K) was generated. The π was also calculated on a sliding window of 100 bases, with a step size of 25 bp to estimate the step-wise diversity across PkDBPαII. The rates of synonymous (*K*s) and non-synonymous (*K*n) mutations were estimated and compared by the Z-test (P <0.05) in MEGA5 using the Nei and Gojobori’s method [11] with Jukes and Cantor correction. In the case of purifying (negative) selection, mutations are usually not advantageous so that *K*n will be less than *K*s (*K*n/*K*s <1). However, in positive selection, non-synonymous mutations can be advantageous and *K*n will exceed *K*s (*K*n/*K*s >1). For testing the neutral theory of evolution, Tajima’s D [12] and Fu and Li’s D and F [13] tests were carried out using DnaSP 5.10.00. In the Fu and Li’s tests, *P. vivax* PvDBPII (GenBank Accession No. M90466) was used as outgroup. The Wright’s F_ST_ fixation index [14] in DnaSP 5.10.00 was used to measure genetic differentiation between the PkDBPαII of North Borneo and Peninsular Malaysia.

## Results

The nested PCR amplification on the human blood samples produced DNA fragments of 1,053 bp in size. The sequence of each fragment was trimmed to 921 bp, as according to the PkDBPαII region described by Singh *et al*. [15]. The trimmed sequence encoded an amino acid sequence of 307 in length. A final total of 49 sequences (GenBank Accession No. KM926563 – KM926611) were obtained.

**Figure 1:**
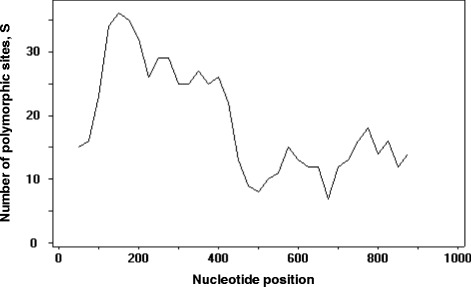
**Nucleotide polymorphism in the PkDBPαII of** ***Plasmodium knowlesi*** **from North Borneo.** Sliding window plot of number of polymorphic sites (S) along the PkDBPαII, generated by using DnaSP ver. 5.10.00 with a window length of 100 bp and step size of 25 bp.

DNA sequence analyses were conducted to determine nucleotide diversity and genetic differentiation. The average number of pair-wise nucleotide differences (K) for the PkDBPαII was 11.261. The overall haplotype diversity (Hd) and nucleotide diversity (π) for the 49 PkDBPαII sequences were 0.999 ± 0.004 and 0.012 ± 0.002, respectively. Detailed analysis of π, with a sliding window plot (window length 100 bp, step size 25 bp), revealed diversity ranged from 0.003 to 0.022. The highest peak of nucleotide diversity was within nucleotide positions 125–250, whereas the most conserved region was within nucleotide positions 625–700 (Figure 1).

Analysis and comparison at the nucleotide level against *P. knowlesi* strain H as reference sequence (GenBank Accession No. M90466) showed mutations at 160 positions among the North Borneo isolates. Fifty-eight of these mutations were synonymous and 102 were non-synonymous. To determine whether natural selection contributed to the diversity in the PkDBPαII, the rate of non-synonymous (*K*n) to synonymous mutations (*K*s) was estimated. *K*n (0.00900) was found lower than *K*s (0.02723) and the *K*n/*K*s ratio was 0.331, suggesting that purifying (negative) selection may be occurring in the PkDBPαII of the North Borneo isolates. Similarly, the Z test (*K*s > *K*n; P <0.05) also indicated purifying selection on PkDBPαII. In the tests of departure of neutrality of selection, the Tajima’s D was −2.459 (P <0.01), indicating expansion in population size and/or purifying selection. This is further supported by the Fu and Li’s D and F tests statistics (−3.713and −3.917, respectively; P <0.02).

**Figure 2:**
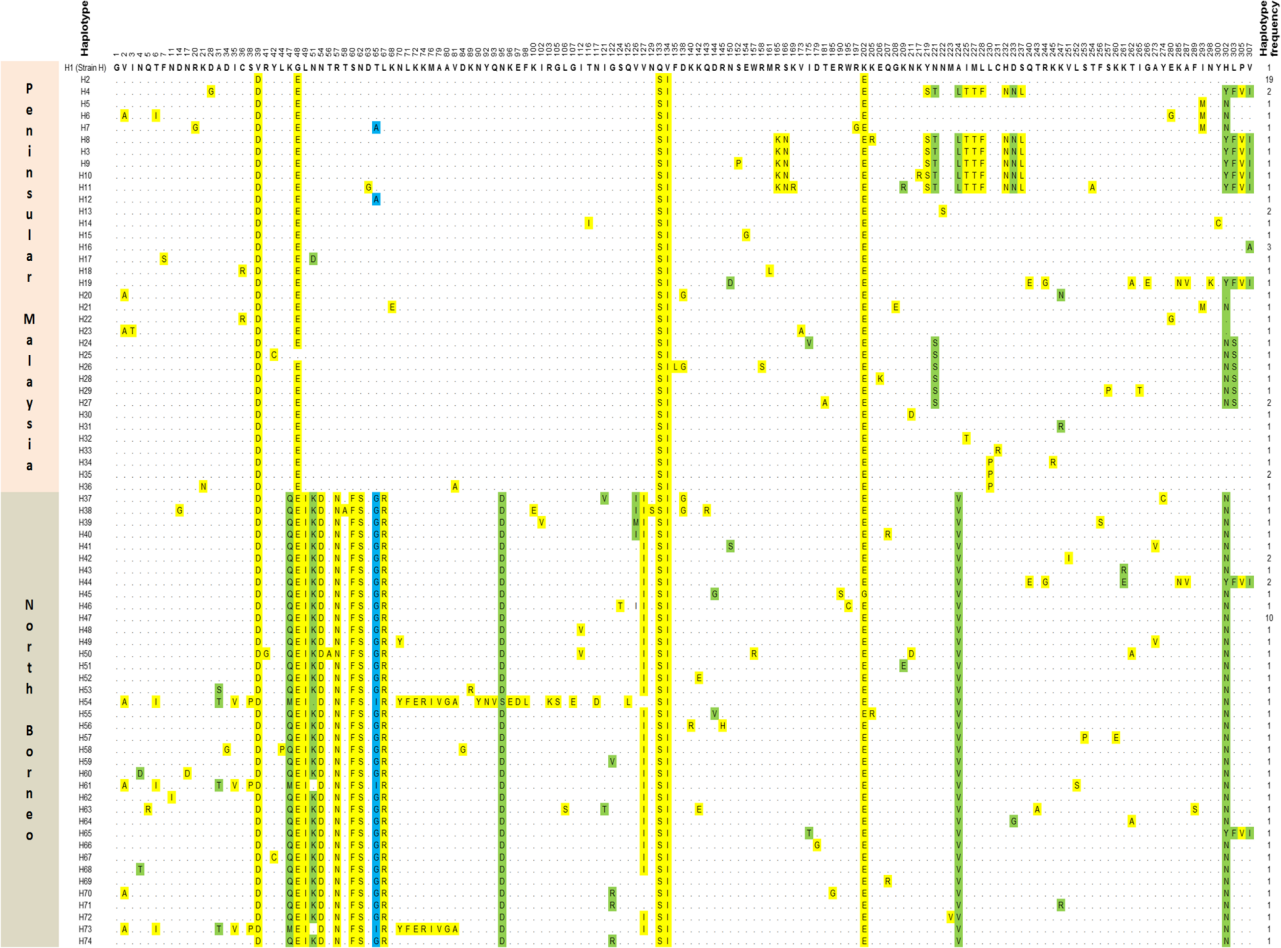
**Amino acid sequence polymorphism in PkDBPαII from Peninsular Malaysia and North Borneo.** Polymorphic amino acid residues are listed for each haplotype. Amino acid residues identical to those of the reference sequence [strain H (haplotype 1)] are marked by dots. Monomorphic, dimorphic and trimorphic amino acid changes are marked in yellow, green and blue shadings, respectively. Total number of sequences for each haplotype is listed in the right panel.

Comparison at the amino acid level against the reference *P. knowlesi* strain H revealed high polymorphism across the PkDBPαII of the North Borneo isolates (Figure 2, bottom half panel). Among the 102 polymorphic sites, 91 were monomorphic change (changed into one amino acid type) and 11 were dimorphic (changed into two amino acid types: positions 4 (N → Q,T), 31(A → S,T), 47 (K → M,Q), 65 (T → G,I), 95 (N → D,S), 121 (I → T,V), 122 (G → R,V), 126 (V → I,M), 144 (D → G,V), 261 (K → E,R), 302 (H → N,Y)). The PkDBPαII amino acid sequences could be categorized into 38 different haplotypes (H37-H74) with haplotype 47 having the highest frequency (10/49). Twelve of the 28 blood samples had mixed haplotype infections (Table 1).

**Figure 3:**
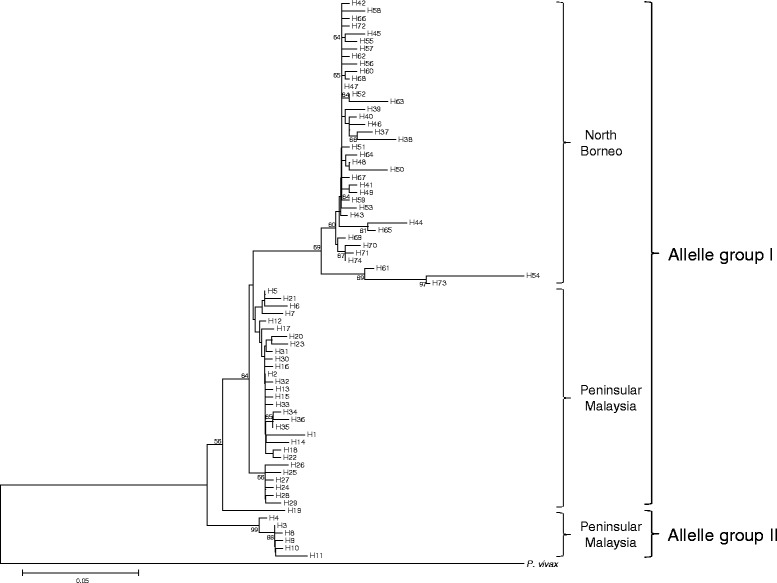
**Phylogenetic tree of PkDBPαII haplotypes.** The Neighbour-Joining method was used to construct the tree, which contains 36 and 38 haplotypes from Peninsular Malaysia (H1-H36) and North Borneo (H37-H74), respectively. *Plasmodium vivax* PvDBPII was used as outgroup. Numbers at nodes indicate percentage support of 1000 bootstrap replicates.

A phylogenetic tree comprising these 38 North Borneo and the 36 Peninsular Malaysia haplotypes reported previously [7], showed interesting features (Figure 3). Overall, the haplotypes are still separated into allele group I and allele group II. All the North Borneo haplotypes are in allele group I. However, they form a sub-group which is distinct from allele group I members from Peninsular Malaysia. The Wright’s F_ST_ value between the PkDBPαII of North Borneo and Peninsular Malaysia was 0.621, indicating high genetic differentiation between these two groups.

## Discussion

The *P. knowlesi* PkDBPαII plays an essential role in the invasion of the parasite by mediating binding with its corresponding receptor, the Duffy protein receptor for chemokines (DARC) on the surface of erythrocytes [16]. The PkDBPαII elicits immune response in humans and therefore has been suggested to be a vaccine candidate antigen [6]. The genetic diversity and haplotype groups of PkDBPαII among Peninsular Malaysia *P. knowlesi* clinical isolates were recently reported [7]. The present study found distinct differences in the PkDBPαII of North Borneo upon comparison with those from Peninsular Malaysia.

Previous studies on *P. vivax* isolates from different geographical regions such as Colombia, South Korea, Papua New Guinea, Thailand, Iran, and Myanmar reported numerous haplotypes and allele groups of PvDBPII [17-22]. Interestingly, some of these PvDBPII haplotypes were grouped with those from outside their geographic origins. For example, haplotypes from Iran were grouped with those from Brazil, Papua New Guines (PNG) and Thailand [21], haplotypes from Myanmar grouped with haplotypes from South Korea [22], and haplotypes from PNG grouped with those from South Korea and Thailand [18,20]. This, however, is not observed in the PkDBPαII in the present study. The phylogenetic analysis (Figure 3) showed a sub-group consisted solely of haplotypes from North Borneo, although these haplotypes were still categorized under allele group I. Geographical separation of Borneo Island from Peninsular Malaysia and subsequent genetic drift of the *P. knowlesi* populations may not be the reason for this unique PkDBPαII separation. This is because other genetic markers studied thus far such as the *P. knowlesi* circumsporozoite protein (*csp*) gene and the small sub-unit ribosomal rRNA (ssu rRNA) do not display such such geographical-based separation [1,23,24].

The PkDBPαII analysed in this study is based on the region defined by Singh *et al*. [15]. In their analysis, 12 C residues (positions 16, 29, 36, 45, 99, 176, 214, 226, 231, 235, 304, 306), which form six disulphide bridges, have been shown to be involved in the folding of PkDBPαII for interaction with DARC. Multiple alignment of the PkDBPαII amino acid sequences (Additional file 1) in this study revealed that these 12 residues were conserved in the PkDBPαII of North Borneo. Apart from these conserved C residues, the Y94, N95, K96, R103, L168, and I175 residues are required for recognition of DARC on human erythrocytes [15]. The multiple sequence alignment showed high conservation of these residues except at position 95. The N (asparagine) residue at this position was substituted with the D (aspartic acid) in the PkDBPαII of North Borneo. However, this N → D substitution may not affect the overall structure and biological function of PkDBPαII, as N is the amide derivative of D.

The PkDBPαII of North Borneo (K = 11.261; Hd = 0.999; π = 0.012) was as diverse as that of Peninsular Malaysia (K = 11.736; Hd = 0.986; π = 0.013). Like the PkDBPαII of Peninsular Malaysia [7], the PkDBPαII of North Borneo was found to be under purifying (negative) selection. A possible reason for this purifying selection is population expansion of *P. knowlesi* in Borneo Island, as evident by the Tajima’s D, as well as the Fu and Li’s D and F tests statistics. Mitochondrial DNA analysis also suggests recent population expansion of *P. knowlesi* in Southeast Asia [25].

Further evidence of difference between the PkDBPαII of North Borneo and Peninsular Malaysia was shown by the Wright’s F_ST_ fixation index, which measures population differentiation due to genetic structure [14]. As a rule of thumb, populations with F_ST_ values of > 0.25 are considered highly differentiated. The F_ST_ obtained in this study was 0.61, indicating extremely high genetic difference between the PkDBPαII of North Borneo and Peninsular Malaysia. The amino acid substitutions in the PkDBPαII, which most likely contribute to this genetic difference, were at positions at positions 47–57, 95 and 224 (Figure 2).

PkDBPαII plays a critical role in the invasion of *P. knowlesi* merozoite into human and monkey erythrocytes. It is crucial for PkDBPαII to conserve its structure for precise interaction with DARC in the invasion process. The discovery in this study of highly differentiated PkDBPαII in North Borneo and Peninsular Malaysia may seem puzzling. However, it has been observed that the *P. vivax* PVDBPII is highly diverse, and in some instances within a population of a particular region [26]. DBPII amino acid residues can be variable and these polymorphisms usually map to non-functional regions of the protein, therefore may serve as a mechanism of immune evasion for the parasite. In such a mechanism, polymorphic residues near the binding site escape binding of host inhibitory antibodies. This protects the crucial functional site on the interacting DBPII domain.

A recent phylogenetic study on the relationships of *Macaca fascicularis*, the natural monkey host of *P. knowlesi*, showed a clear separation between Borneo’s and Peninsular Malaysia’s populations [27]. This phylogeny was based on the cytochrome b gene sequences of the monkeys. It is, therefore, worthwhile in future studies to determine whether a similar genetic separation occurs in the DARC of the monkey populations, and to associate it with the PkDBPαII haplotype groups observed in this study.

## Conclusions

This study is the first to report the genetic diversity and natural selection of PkDBPαII of *P. knowlesi* from Borneo Island. The PkDBPαII haplotypes found in this study were distinct from those from Peninsular Malaysia. This difference may not be attributed to geographical separation because other genetic markers studied thus far such as the *P. knowlesi* circumsporozoite protein gene and small subunit ribosomal RNA do not display such differentiation. Immune evasion may possibly be the reason for the differentiation.

## Additional file

Additional file 1:
**Full amino acid sequence alignment of PkDBPαII from Peninsular Malaysia and North Borneo.** Amino acid residues identical to those of the reference sequence (strain H) are indicated by dots. The twelve conserved cysteine (C) residues are marked in yellow. The conserved Y94, N95, K96, R103, L168 and I175 residues required for recognition of DARC on human erythrocytes are highlighted in green. Note that the N9 residue was substituted by D95 in the North Borneo sequences.
